# FLZ Alleviates the Memory Deficits in Transgenic Mouse Model of Alzheimer’s Disease via Decreasing Beta-Amyloid Production and Tau Hyperphosphorylation

**DOI:** 10.1371/journal.pone.0078033

**Published:** 2013-11-04

**Authors:** Xiu-Qi Bao, Ning Li, Tao Wang, Xiang-Chen Kong, Wen-Jiao Tai, Hua Sun, Dan Zhang

**Affiliations:** 1 State Key Laboratory of Bioactive Substance and Function of Natural Medicine, Institute of Materia Medica, Chinese Academy of Medical Sciences and Peking Union Medical College, Beijing, China; 2 Beijing Municipal Corps Hospital of Chinese People’s Armed Police Force, Beijing, China; 3 School of Medicine, Shenzhen University, Beijing, China; New York State Institute for Basic Research, United States of America

## Abstract

Alzheimer’s disease (AD) is the most common cause of dementia worldwide and mainly characterized by the aggregated β-amyloid (Aβ) and hyperphosphorylated tau. FLZ is a novel synthetic derivative of natural squamosamide and has been proved to improve memory deficits in dementia animal models. In this study, we aimed to investigate the mechanisms of FLZ’s neuroprotective effect in APP/PS1 double transgenic mice and SH-SY5Y (APPwt/swe) cells. The results showed that treatment with FLZ significantly improved the memory deficits of APP/PS1 transgenic mice and decreased apoptosis of SH-SY5Y (APPwt/swe) cells. FLZ markedly attenuated Aβ accumulation and tau phosphorylation both *in vivo* and *in vitro*. Mechanistic study showed that FLZ interfered APP processing, i.e., FLZ decreased β-amyloid precursor protein (APP) phosphorylation, APP-carboxy-terminal fragment (APP-CTF) production and β-amyloid precursor protein cleaving enzyme 1 (BACE1) expression. These results indicated that FLZ reduced Aβ production through inhibiting amyloidogenic pathway. The mechanistic study about FLZ’s inhibitory effect on tau phosphorylation revealed t the involvement of Akt/glycogen synthase kinase 3β (GSK3β) pathway. FLZ treatment increased Akt activity and inhibited GSK3β activity both *in vivo* and *in vitro*. The inhibitory effect of FLZ on GSK3β activity and tau phosphorylation was suppressed by inhibiting Akt activity, indicating that Akt/GSK3β pathway might be the possible mechanism involved in the inhibitory effect of FLZ on tau hyperphosphorylation. These results suggested FLZ might be a potential anti-AD drug as it not only reduced Aβ production via inhibition amyloidogenic APP processing pathway, but also attenuated tau hyperphosphoylation mediated by Akt/GSK3β.

## Introduction

Alzheimer’s disease (AD) is the most common cause of dementia worldwide. The hallmarks of AD are mainly characterized by senile plaques (SPs) and neurofibrillary tangles (NFTs), which consist of aggregated β-amyloid protein (Aβ) and hyperphosphorylated tau protein (p-tau). Aβ peptides are generated by successive proteolysis of β-amyloid precursor protein (APP), a large transmembrane glycoprotein, which is initially cleaved by the β-amyloid precursor protein cleaving enzyme 1 (BACE1) and subsequently by γ-secretase in the transmembrane domain [1]. Aggregated Aβ plays a pivotal role in the pathogenesis of AD [2]. After penetrating neuronal membrane, Aβ aggregates and destroys cell membrane [3], thus inducing neuronal loss and memory deficits [4], [5]. Moreover, Aβ alters cellular metabolism and triggers downstream tau hyperphosphorylation/tangle formation, which plays a major role in the onset of cognitive decline and tau pathology. Aberrant hyperphosphorylated tau is reported to lose its ability to bind and stabilize microtubules, resulting in destabilization of the cytoskeleton and perturbation of axonal transport [6].

**Figure 1:**
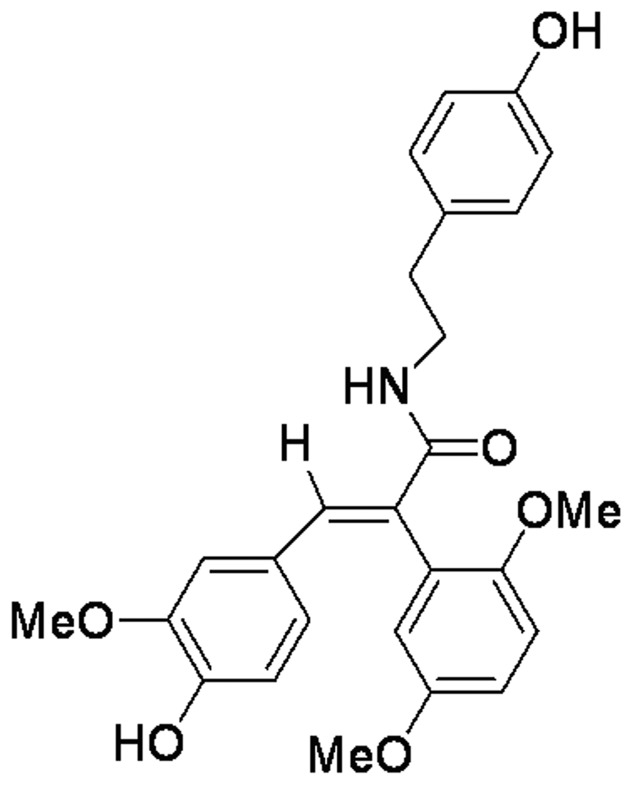
Chemical structure of FLZ, N-[2-(4-hydroxy-phenyl)-ethyl] -2-(2,5-dimethoxy-phenyl)-3-(3-methoxy-4-hydroxy-phenyl)-acrylamide.

Current drugs for AD treatment such as acetylcholinesterase inhibitor and NMDA antagonist show limited benefits to most AD patients [7]. Therefore, there is an urgent need for novel therapeutic strategies that could halt the disease process and are suitable for long-term clinical management. A new viewpoint has emerged that the treatment of AD can be more effective at specific targets, such as oligomeric Aβ and tau hyperphosphorylation [7]. Therefore, exploring compounds which prevent Aβ accumulation or tau hyperphosphorylation has been the main focus of the drug development in recent years. We have previously reported that FLZ (Fig. 1), a novel synthetic cyclic analogue of natural squamosamide from the leaves of *Anona squamosa*, has neuroprotective effects on the *in vivo* and *in vitro* AD models. FLZ improved memory deficits of natural aging mice [8], demential model mice induced by Aβ_25–35_ (Fang & Liu, 2006) and D-galactose plus NaNO_2_ [9]. FLZ also markedly prevented neuronal apoptosis induced by Aβ_25∼35_ [10]. Pharmacokinetic study indicated that FLZ could cross the blood-brain barrier and reach cortex and hippocampus [11]. These previous studies suggested FLZ had potential therapeutic effect on AD-associated pathologies. However, the underling mechanism of FLZ’s therapeutic effect on AD models has not yet been ascertained.

The present study was designed to investigate the beneficial effects of FLZ on learning and memory deficits of AD model mice and the possible mechanisms. Consistent with previous studies [12], [13], APP/PS1 transgenic mice demonstrated memory impairment, Aβ overproduction and tau hyperphosphorylation. Using APP/PS1 transgenic mice and SH-SY5Y (APP wt/swe) cells, we found that FLZ significantly improved learning and memory ability of mice, decreased Aβ production and tau phosphorylation. Mechanistic study indicated that FLZ attenuated Aβ production by inhibiting APP phosphorylation and BACE1 expression, inhibited tau hyperphosphorylation through regulating Akt/glycogen synthase kinase 3β (GSK3β) pathway.

## Materials and Methods

### Regents and Antibodies

FLZ, a white powder with 99% purity, was synthesized by the Department of Pharmaceutical Chemistry, Institute of Materia Medica, Chinese Academy of Medical Science. It was suspended in 0.5% (w/v) sodium carboxymethyl cellulose (CMC-Na) for oral administration and dissolved in dimethylsulfoxide (DMSO) for *in vitro* use. Dulbecco’s modified Eagle medium, Nutrient Mixture F-12 (DMEM/F-12), fetal bovine serum and G418 were purchased from Gibico (Grand Island, NY, USA). β-amyloid ELISA kit was product of Invitrogen. Phosphorylated tau at Ser396, Ser404 and Thr231, phosphorylated Akt, Akt and insulin-degrading enzyme (IDE) antibodies were purchased from Signalway. Phosphorylated tau at Ser202/Thr205 (clone AT8) antibody was purchased from Pierce Biotechnology. APP, APP C-terminal fragment (APP-CTF) and Aβ (clone 6E10) antibodies were from Abcam. BACE1 antibody was obtained from Millipore. Phosphorylated APP (Thr668) antibody and Ly294002 were purchased from Cell Signaling. Phosphorylated GSK3β, GSK3β antibodies were from Santa Cruz Biotechnology. Caspase-3 assay kit was purchase from Sigma.

### Ethics Statement

All experimental procedures were approved by the Institutional Animal Care and Use Committee of the Peking Union Medical College and performed in accordance with the National Institutes of Health guide for the care and use of laboratory animals. All efforts were made to minimize animal suffering.

### Animal and Treatment

Male B6C3 mice expressing Swedish mutant AβPP695 and the exon-9 deletion mutant PS1 (APPswe/PS1Δ9) were supplied by the Animal Center of Chinese Academy of Medical Sciences. Mice were maintained in a 12-hours light/dark cycle at 24°C in a relative humidity of 60% room, and received food and water *ad libitum*. FLZ 150 mg/kg were orally administered to APP/PS1 double-transgenic mice and the WT mice were orally given 0.5% CMC-Na at 5 months of age for continued 20 weeks.

### Cell Culture and Treatment

SH-SY5Y cells stably expressed wild-type APP and Swedish mutant APP (APPwt/swe) were gifts from Prof. Bao-Lu Zhao from Institute of Biophysics, Chinese Academy of Sciences. SH-SY5Y cells transfected with empty pcDNA3.1neo vector was used as negative control. The cells were maintained in DMEM/F-12 medium supplemented with 10% fetal bovine serum, 100 U/ml penicillin, 100 µg/ml streptomycin and 200 µg/ml G418 at 37°C in a humidified 5% CO_2_/95% air incubator. When the confluency reached 80%, the cells were switched to a “stimulating medium” containing 50% DMEM, 50% Opti-MEM, 0.5% FBS, 200 µg/ml G418 and 10 mM butyric acid sodium salt for 12 h to induce the transgene expression. FLZ (0.1, 1 and 10 µM), Ly294002 10 µM combined with FLZ 10 µM or alone were incubated with cells for 24 h, and then the cells were harvested to test the related biomarkers.

### Morris Water Maze Test

Spatial learning and memory o mice was assessed in the Morris water maze (Institute of Materia Medica, Chinese Academy of Medical Sciences and Peking Union Medical College, Beijing, China) after the mice had received FLZ for 20 weeks (n = 10 in each group). The Morris water maze consisted of a circular pool (120 cm diameter and 40 cm deep) filled with nontoxic opaque water with an escape platform (10 cm diameter) hidden beneath the water (2 cm). The water temperature was maintained at 23±1°C. At the beginning of each trial, the mouse was placed into the water facing the wall of the pool at one of the four quadrants. Although the starting point was randomly selected, the protocol was fixed at the beginning of each trial and was maintained throughout all four acquisition trials. Each mouse was allowed 120 s to find and mount the platform. When the mouse found the platform, it was kept on the platform for 30 s. If the mouse failed to find the platform within 120 s, it was placed on the platform where it remained for 30 s. Each mouse was given 4 trials per day, with an interval of 1 h. The time to find the platform (escape latency), the total distance traveled, and the swim speed of the animals were recorded by video tracking software. The mice were then towel dried and placed in a cage with a heating pad underneath until dry and returned to their home cage. The training session was conducted for 5 consecutive days in which the platform was never moved. The latency to escape was calculated as the average time to find the platform of the 4 trials within one day.

Memory retention was evaluated on the 6th day with probe trial in which the platform was removed. The mice were put into the pool and allowed to swim freely in the pool for 120 s. the number of crossings of the platform and the time spent in the target quadrant were recorded.

### Immunohistochemical Analysis

Five mice from each group were anesthetized with pentobarbital and perfused transcardially with saline and then with cold 4% paraformaldehyde in 0.1 M phosphate buffer, pH 7.4. Brains were then removed and post-fixed for 4 h in the same fixative. The fixed brains were washed and cryoprotected in the same buffer containing 20% sucrose, and finally sectioned into 40 µm sections on a freezing microtome. Every sixth serial section was selected and processed for TH immunostainning. Coronal sections through the hippocampus were processed for Aβ immunohistochemistry. Briefly, after incubation for 1 h in 10% normal swine serum with 0.25% TritonX100 in 0.02 M potassium-phosphate-buffered saline containing 1% bovine serum albumin (KPBS-BSA), sections were incubated with the primary antibody (mouse monoclonal antiserum to Aβ, 1∶500 in KPBS-BSA containing 2% normal swine serum and 0.25% Triton X100) and then incubated first with the corresponding biotinylated secondary antibody and subsequently for 90 min with avidin-peroxidase. Finally, the labeling was visualized with 0.04% hydrogen peroxidase and 0.05% 3,3′-diaminobenzidine (DAB). The sections were observed with light microscopy (NIKON E600, Japan), and the intensity of stained area in each group was analyzed by Image-Pro plus system (Media Cybernetics, Silver Spring, MD). All evaluations were done by a researcher blind to the experimental design.

### ELISA Assay

The supernatants of cells were harvested, centrifuged and 10× concentrated in presence of a protease inhibitors cocktail (2.5 mM EDTA, 10 µM leupeptin, 1 µM peptastin, 1 mM phenylmethylsulfonyl fluoride) cocktail using Centricon (Amicon) with a cutoff value of 3 kDa. For the measurement of intracellular Aβ, approximately 10^7^ cells were scraped in ice cold PBS. Cell pellets were solubilized in 300 ml of 70% formic acid. Formic acid-solubilized cell pellets were cleared by centrifuging at 16,000 g for 5 min at 4°C, and the supernatants were centrifuged at 21,000 g for 20 min at 4°C. The supernatants were vacuum dried and resulting pellet was resuspended in 1 ml of alkaline carbonate buffer (2% Na_2_CO_3_, 0.1N NaOH) and centrifuged at 16,000 g for 3 min at 4°C. Aβ production was measured by a sensitive fluorescence based sandwich ELISA assay using a kit (Human β-Amyloid 1–40 Colorimetric Immunoassay Kit) according to the manufacturer’s instructions.

### Western Blot Assay

Mouse hippocampus (four mice from each group) and cells were lysed in nondenaturing lysis buffer. The lyses were then centrifuged at 12,000 g for 15 min at 4°C, the supernatants were mixed with loading buffer and boiled for 5 min. Protein concentration was measured by Bradford protein assay. Samples containing 30 µg of protein per lane were separated by 10% sodium dodecyl sulfate–polyacrylamide gel electrophoresis (SDS-PAGE) and transferred to PVDF membranes (For Aβ assay, 16% Tris-Tricine gel was used.). The membranes were blocked in 5% skim milk-TBST (20 mM Tris-HCL, pH 7.5, 500 mM NaCl, 0.1% Tween 20) for 1 h, then respective primary antibodies were added in the same milk and incubated overnight at 4°C, and then incubated with horseradish peroxidase-conjugated secondary antibody in TBST for 2 h at room temperature. The blot was developed with LAS3000 chemiluminescence system (Fujifilm, Tokyo, Japan), and the densities of the bands were determined using Gel-Pro Analyzer 4.0 software.

### Flow Cytometry Assay

The apoptosis rate of cells was assayed by flow cytometry according to the manufacture’s instruction. Briefly, the cells were harvested and washed twice with PBS, followed by resuspending in 500 µl binding buffer, and then incubated with 5 µl Annexin V and 5 µl Propidium Iodide (PI) for 15 min in the dark at room temperature. Then 1×10^4^ cells were measured with a flow cytometer using the Cell Quest software.

### Caspase-3 Activity Determination

Caspase-3 activity was determined according to the manufacturer’s protocol. In brief, cell lysates were prepared by lysis buffer. Caspase-3 activity was determined by monitoring proteolysis of the colorimetric substrates. Ac-DEVD-p-nitroaniline was used as colorimetric p-nitroaniline linked substrate. The whole-cell lysate was added to a buffer containing 200 µM substrate. After 1.5 h of incubation, the cleavage of the peptide by the caspase was quantified spectrophotometrically at 405 nm in a 96 well plate. The unit of the optical density was converted to nmols of p-nitroaniline using a standard curve generated with free p-nitroaniline.

### Statistical Analysis

Data were expressed as means ±SD. MANOVA was used for repeated measurement of escape latency data, using a general linear model (GLM) in SPSS 13.0. Other data were analyzed by one-way ANOVA followed by Dunnett’s post hoc test. *P*<0.05 was considered to be statistically significant.

## Results

### FLZ Reduced Spatial Learning and Memory Deficits of APP/PS1 Mice

**Figure 2:**
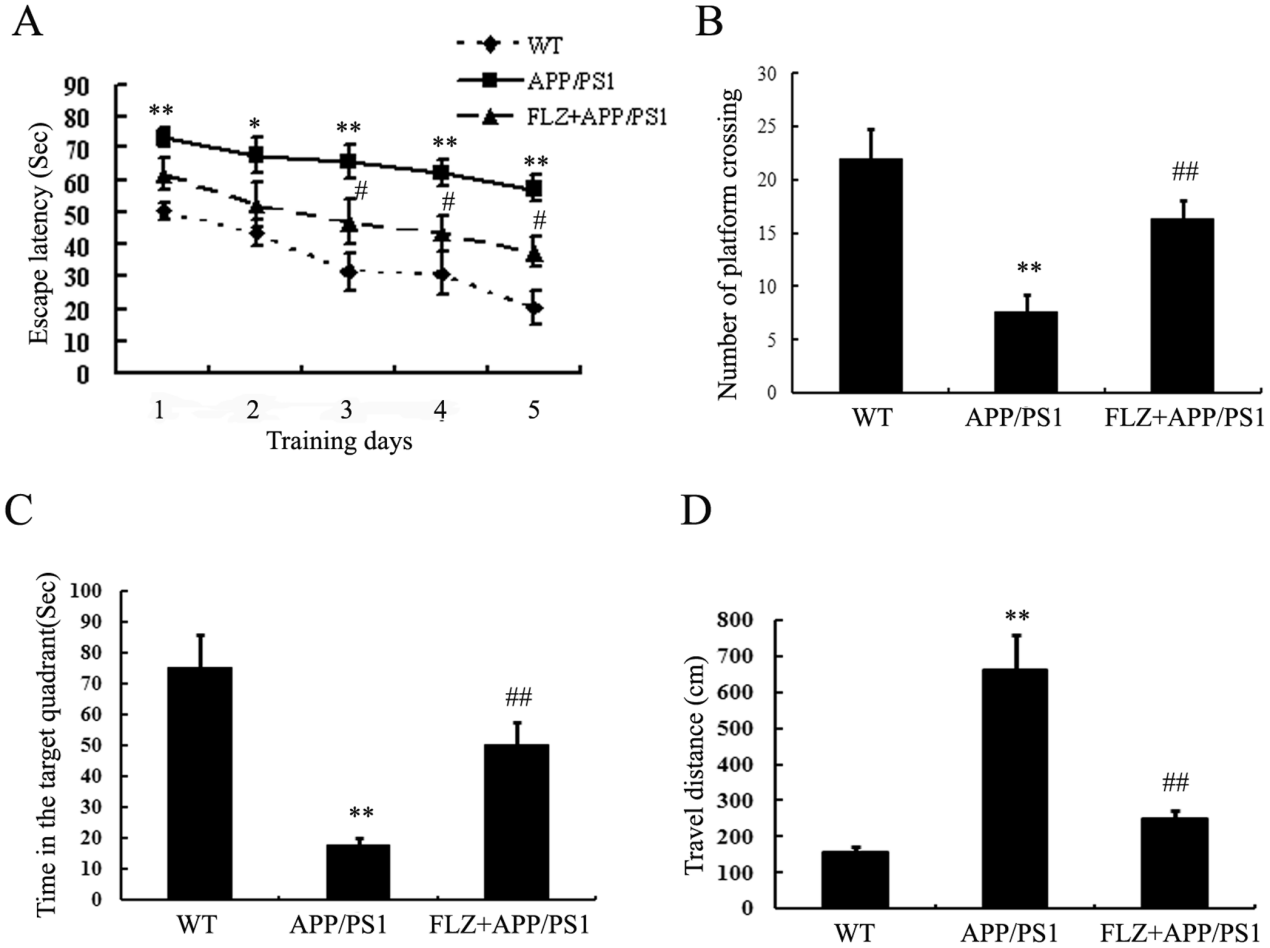
FLZ ameliorated learning and memory deficits of APP/PS1 mice. APP/PS1 double transgenic mice were orally treated with FLZ 150 mg/kg for 20 weeks, and the learning and memory ability was accessed by passage water maze test. (A) The latencies of mice to find the destination. (B) Number of platform crossing. (C) Time in the target quadrant. (D) Travel distance. Results were expressed as mean±SD. *P<0.05,**P<0.01 *vs,* WT mice, ^#^ *P*<0.05 *vs*. APP/PS1 transgenic mice. n = 10 in each group.

FLZ treatment started when the APP/PS1 double-transgenic mice were 5 months old and exhibited memory deficits (Fig. S1 in File S1). After FLZ was administrated daily for consecutive 20 weeks, the mice were subjected for Morris water maze test. As shown in Fig. 2, APP/PS1 mice showed significantly longer escape latencies than the wide-type (WT) mice (*P*<0.01), confirming the impaired spatial learning in the APP/PS1 mice. FLZ treatment significantly decreased the escape latency at the 3^rd^, 4^th^ and 5^th^ day of the test (*P*<0.05) (Fig. 2). In the probe test, the number of APP/PS1 mice crossing the platform and the time spent in the target quadrant remarkably decreased compared with WT mice, and the travel distances were much longer than those of WT mice. FLZ treatment significantly increased the number of platform crossing (1.16-fold increase, *P*<0.01) and the time spent in the target quadrant (1.8-fold increase, *P*<0.01), and the travel distance was decreased by FLZ treatment (62.18% decrease, *P*<0.01). There were no differences of swimming speed among the three groups of mice. Besides, the body weights of mice in all the groups were at the same level during the test (Fig. S2 in File S1). These data indicated that FLZ had beneficial effects on the learning and memory lesion of APP/PS1 transgenic mice.

### FLZ Protected SH-SY5Y (APPwt/swe) Cells from Apoptosis

**Figure 3:**
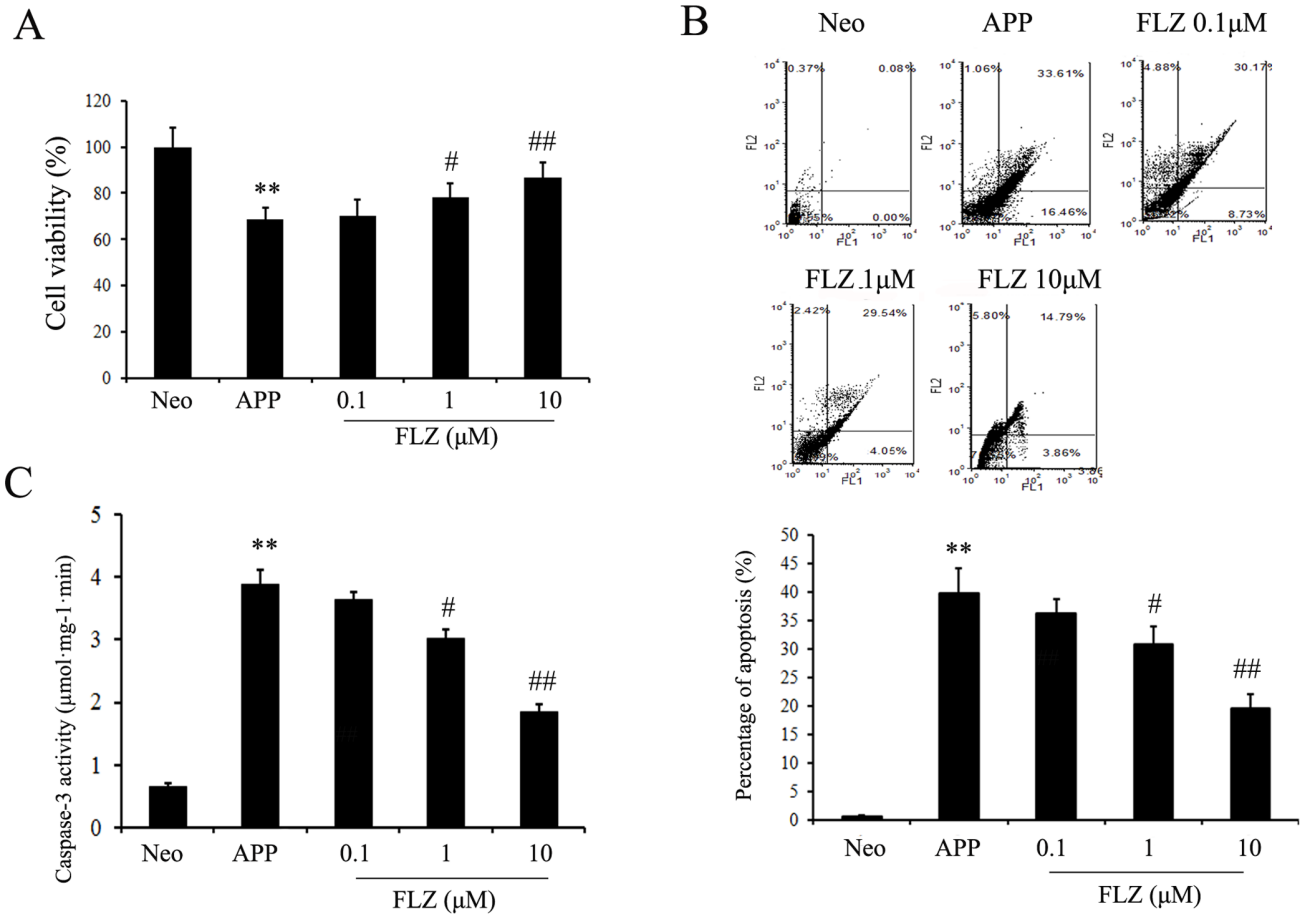
FLZ protected SH-SY5Y (APPwt/swe) cells from apoptosis. SH-SY5Y (APPwt/swe) cells were grown in “stimulating medium” containing 50% DMEM, 50% Opti-MEM, 0.5% FBS, 200 µg/ml G418 and 10 mM butyric acid sodium salt for 12 h to induce the transgene expression. FLZ (0.1, 1 and 10 µM) were incubated with cells for 24 h. (A) Cell viability of SH-SY5Y (APPwt/swe) cells. (B) Apoptosis of SH-SY5Y (APPwt/swe) cells measured by flow cytometry with Annexin-V/PI staining. Bar chart is the statistical of the sum of early and late cell apoptosis. (C) Caspase-3 activity of SH-SY5Y (APPwt/swe) cells. Results were expressed as mean ± SD from 6 independent experiments. **P<0.01 *vs*. Neo SH-SY5Y cells, ^#^ *P*<0.05, ^##^ *P*<0.01 *vs*. solvent-treated SH-SY5Y (APPwt/swe) cells.

The neuroprotective effects of FLZ were also observed in SH-SY5Y (APPwt/swe) cells. As shown in Fig. 3, FLZ markedly increased cell viability (FLZ 1 µM: 14.25% increase, *P*<0.05; FLZ 10 µM: 26.71% increase, *P*<0.01) of SH-SY5Y (APPwt/swe) cells. The apoptosis was analyzed by flow cytometry with Annexin-V/PI staining to determine the percentages of early apoptosis (AnnexinV^+^/PI^−^) and late apoptosis (AnnexinV^+^/PI^+^) of cells. The results showed that FLZ reduced both early and late apoptosis of cells in a dose-dependent manner (FLZ 1 µM: 22.66% decrease, *P*<0.05; FLZ 10 µM: 50.81% decrease, *P*<0.01). The results of flow cytometry were confirmed by the measurement of caspase-3 activity, which showed that FLZ markedly reduced caspase-3 activity (FLZ 1 µM: 22.62% decrease, *P*<0.05; FLZ 10 µM: 52.18% decrease, *P*<0.01) of SH-SY5Y (APPwt/swe) cells, indicating that FLZ protected neurons from apoptosis stimulated by Aβ (Fig. 3).

### FLZ Inhibited Aβ Production in APP/PS1 Mice and SH-SY5Y (APPwt/swe) Cells

**Figure 4:**
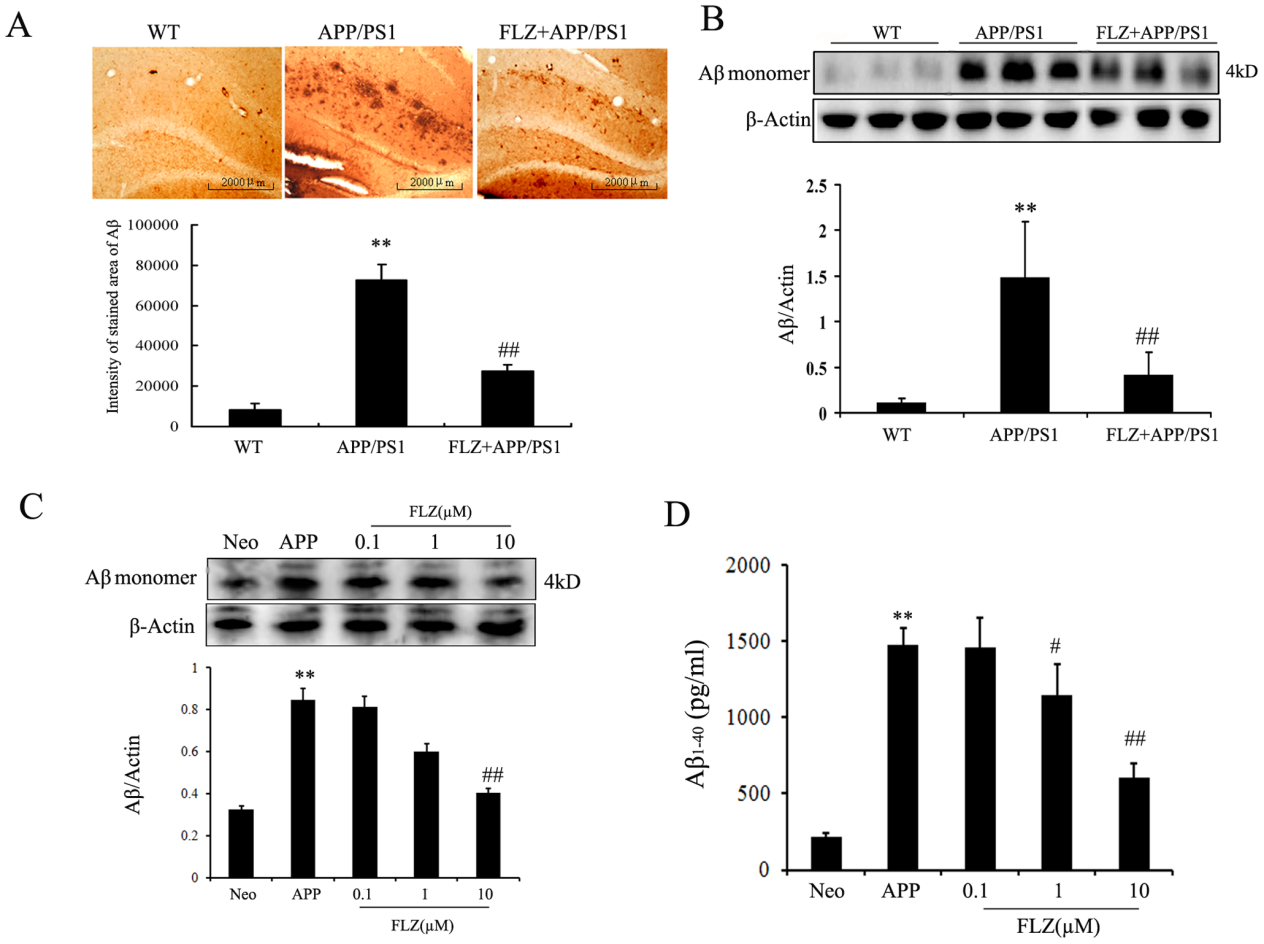
FLZ inhibited Aβ production in APP/PS1 mice and SH-SY5Y (APPwt/swe) cells. APP/PS1 double transgenic mice were orally treated with FLZ 150 mg/kg for 20 weeks. SH-SY5Y (APPwt/swe) cells were grown in “stimulating medium” containing 50% DMEM, 50% Opti-MEM, 0.5% FBS, 200 µg/ml G418 and 10 mM butyric acid sodium salt for 12 h to induce the transgene expression. FLZ (0.1, 1 and 10 µM) were incubated with cells for 24 h. (A) Immunohistochemistry of Aβ deposits in hippocampus. Representative sections of hippocampus from 5 mice were shown. Scale bar: 500 µm. (B) Western blot assay of Aβ expression in hippocampus. A representative immunoblot from 4 mice was shown. Results were expressed as mean±SD. **P<0.01 *vs*. WT mice; ^#^ *P*<0.05, ^##^ *P*<0.01 *vs*. APP/PS1 mice. (C) Western blot assay and (D) ELISA assay of Aβ_1–40_ in SH-SY5Y (APPwt/swe) cells. The results were obtained from 4–6 independent experiments. Results were expressed as mean±SD. **P<0.01 *vs*. Neo SH-SY5Y cells, ^#^ *P*<0.05, ^##^ *P*<0.01 *vs*. solvent-treated SH-SY5Y (APPwt/swe) cells.

The effect of systemic administration of FLZ on Aβ production in APP/PS1 double-transgenic mice was tested. Consistent with previous studies [12], [13], immunohistochemical assay showed that there were large areas of Aβ deposits in hippocampus of APP/PS1 mice. FLZ treatment significantly reduced the areas of Aβ deposits in hippocampus (62.17% decrease, *P*<0.01) (Fig. 4A). We also found that FLZ decreased Aβ deposits in cortex of APP/PS1 mice (Fig. S3 in File S1). Similar data were obtained in Western blot assay, which showed the increased amount of Aβ in hippocampus of APP/PS1 mice and the therapeutic effect of FLZ (71.62% decrease, *P*<0.01) (Fig. 4B).

The above *in vivo* data were confirmed in the following *in vitro* experiment, in which SH-SY5Y (APPwt/swe) cells were used. This cell line stably overexpresses the Swedish mutant form of human APP and overproduces Aβ. Western blot assay and ELISA assay were applied to measure the intracellular and extracellular Aβ levels, respectively. The results showed that both intracellular and extracellular Aβ levels significantly increased in SH-SY5Y (APPwt/swe) cells. FLZ 10 µM significantly reduced intracellular Aβ production (73.33% decrease, *P*<0.05), FLZ 1 µM and 10 µM markedly decreased levels of extracellular Aβ (FLZ 1 µM: 44.56% decrease, *P*<0.05; FLZ 10 µM: 63.93% decrease, *P*<0.01) (Fig. 4 C, D). The above data indicated that FLZ potentially inhibited the production of Aβ.

### FLZ Decreased the Expression of Phospho-APP and APP-CTF

**Figure 5:**
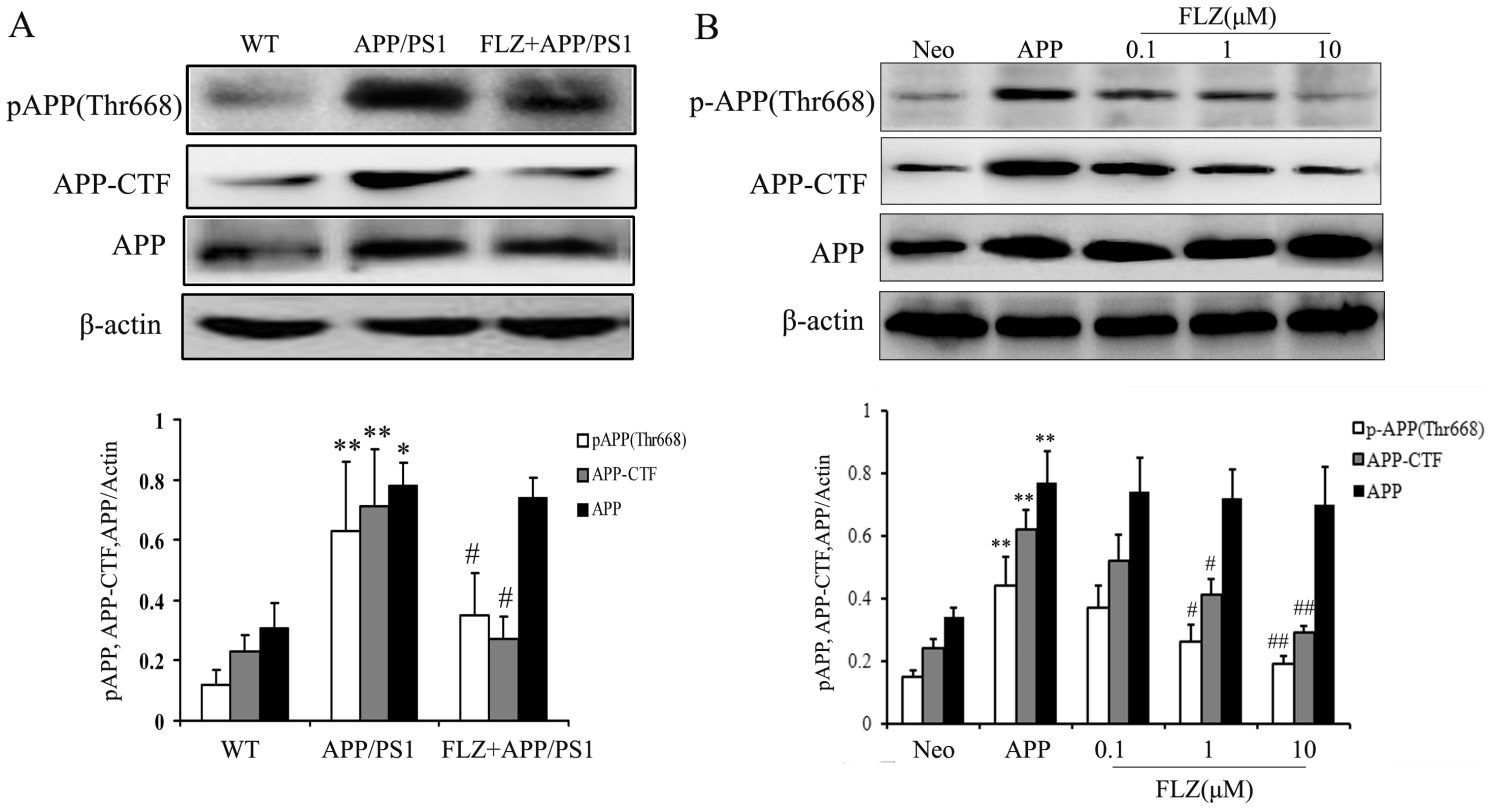
FLZ decreased the expression of phospho-APP and APP-CTF. APP/PS1 double transgenic mice were orally treated with FLZ 150 mg/kg for 20 weeks. SH-SY5Y (APPwt/swe) cells were grown in “stimulating medium” containing 50% DMEM, 50% Opti-MEM, 0.5% FBS, 200 µg/ml G418 and 10 mM butyric acid sodium salt for 12 h to induce the transgene expression. FLZ (0.1, 1 and 10 µM) were incubated with cells for 24 h. Antibodies against p-APP, APP-CTF and APP were subjected to Western blot assay. A representative immunoblot from 4 mice was shown. Results were expressed as mean ±SD. (A) Western blot assay of APP, phospho-APP and APP-CTF in hippocampus of APP/PS1 mice. *P<0.05,**P<0.01 *vs*. WT mice; ^#^ *P*<0.05 *vs*. APP/PS1 mice. (B) Western blot assay of APP, phospho-APP and APP-CTF in SH-SY5Y (APPwt/swe) cells. **P<0.01 *vs*. Neo SH-SY5Y cells, ^#^ *P*<0.05, ^##^ *P*<0.01 *vs*. solvent-treated SH-SY5Y (APPwt/swe) cells.

Aβ is a proteolytic product of APP, and phosphorylation at Thr668 of APP facilitates its processing towards Aβ. So we measured the total APP and phospho-APP (Thr668) levels in the hippocampus of APP/PS1 mice and SH-SY5Y (APPwt/swe) cells. The results showed that APP and phospho-APP (Thr668) levels markedly increased in the hippocampus of APP/PS1 mice and SH-SY5Y (APPwt/swe) cells. FLZ treatment did not affect the level of total APP, but significantly reduced APP phosphorylation at Thr668 both *in vivo* (44.44% decrease, *P*<0.05) and *in vitro* (FLZ 1 µM: 52.94% decrease, *P*<0.05; FLZ 10 µM: 73.53% decrease, *P*<0.01) (Fig. 5), which indicated the interference of FLZ on APP processing.

The phospho-APP is cleaved by BACE1, and produces membrane-bound C-terminal fragment (CTF). The APP-CTF is then digested by γ-secretase within the transmembrane region, which generates small secreted peptides, including the pathogenic Aβ. So the increased level of APP-CTF will lead to increased production of Aβ. To test whether FLZ inhibited APP-CTF production, APP/PS1 mouse hippocampus was subjected to Western blot analysis. As shown in Fig. 5A, a significant decrease of APP-CTF expression was observed in FLZ-treated APP/PS1 mice (61.97% decrease, *P*<0.01). APP-CTF expression was also reduced by FLZ treatment in SH-SY5Y (APPwt/swe) cells (FLZ 1 µM: 40.48% decrease, *P*<0.05; FLZ 10 µM: 69.05% decrease, *P*<0.01) (Fig. 5B). The above results indicated that the inhibitory effects of FLZ on Aβ production may be through attenuating APP phosphorylation and APP-CTF production.

### FLZ Decreased the Expression of BACE1

**Figure 6:**
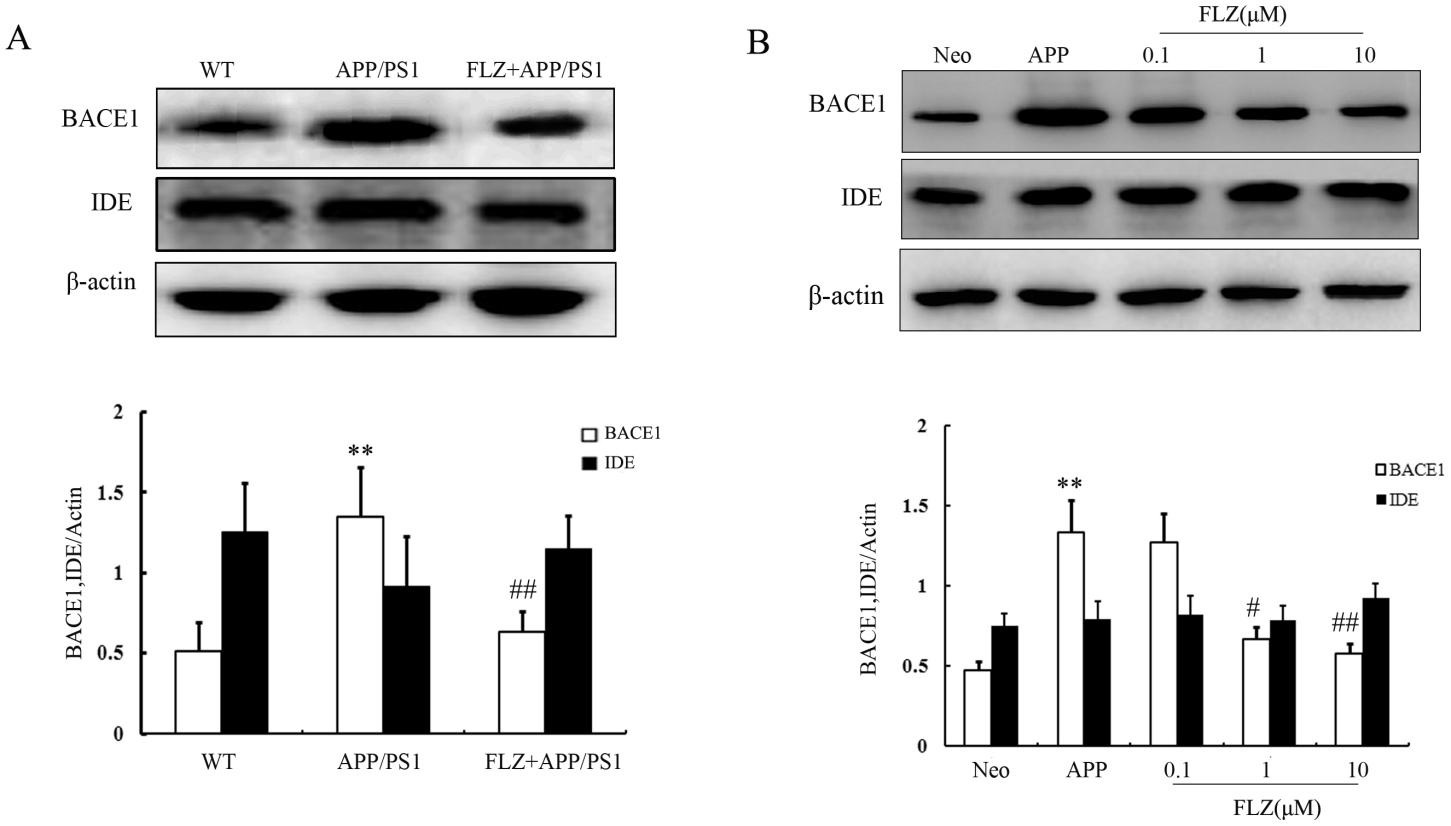
FLZ decreased the expression of BACE1 and did not affect the expression of IDE. APP/PS1 double transgenic mice were orally treated with FLZ 150 mg/kg for 20 weeks. SH-SY5Y (APPwt/swe) cells were grown in “stimulating medium” containing 50% DMEM, 50% Opti-MEM, 0.5% FBS, 200 µg/ml G418 and 10 mM butyric acid sodium salt for 12 h to induce the transgene expression. FLZ (0.1, 1 and 10 µM) were incubated with cells for 24 h. (A) Western blot assay of BACE1 and IDE in the hippocampus of APP/PS1 mice treated with FLZ. A representative immunoblot from 4 mice was shown. Results are expressed as mean±SD. *P<0.05 *vs*. WT mice; ^#^ *P*<0.05 *vs*. APP/PS1 mice. (B) Western blot assay of BACE1 and IDE in SH-SY5Y (APPwt/swe) cells treated with FLZ. A representative immunoblot from four independent experiments was shown. Results were expressed as mean ± SD. **P<0.01 *vs*. Neo SH-SY5Y cells, ^#^ *P*<0.05, ^##^ *P*<0.01 *vs*. solvent-treated SH-SY5Y (APPwt/swe) cells.

We further examined the effects of FLZ on the enzymes that contribute to the production of Aβ. BACE1 is the key enzyme that is responsible for APP processing and Aβ production. In the present study, it was observed that FLZ treatment markedly decreased the expression of BACE1 in APP/PS1 mouse hippocampus (53.33% decrease, *P*<0.01) and SH-SY5Y (APPwt/swe) cells (FLZ 1 µM: 49.62% decrease, *P*<0.05; FLZ 10 µM: 56.39% decrease, *P*<0.05) (Fig. 6).

The extracellular degradation of Aβ is carried out primarily principally by insulin degrading enzyme (IDE). We then tested the effect of FLZ on IDE expression. The present results showed that FLZ did not affect the expression of IDE. These data suggested that FLZ decreased Aβ level through inhibiting Aβ production without affecting its degradation (Fig. 6).

### FLZ Attenuated Tau Phosphorylation at Ser396, Ser202/Thr205 and Thr231

**Figure 7:**
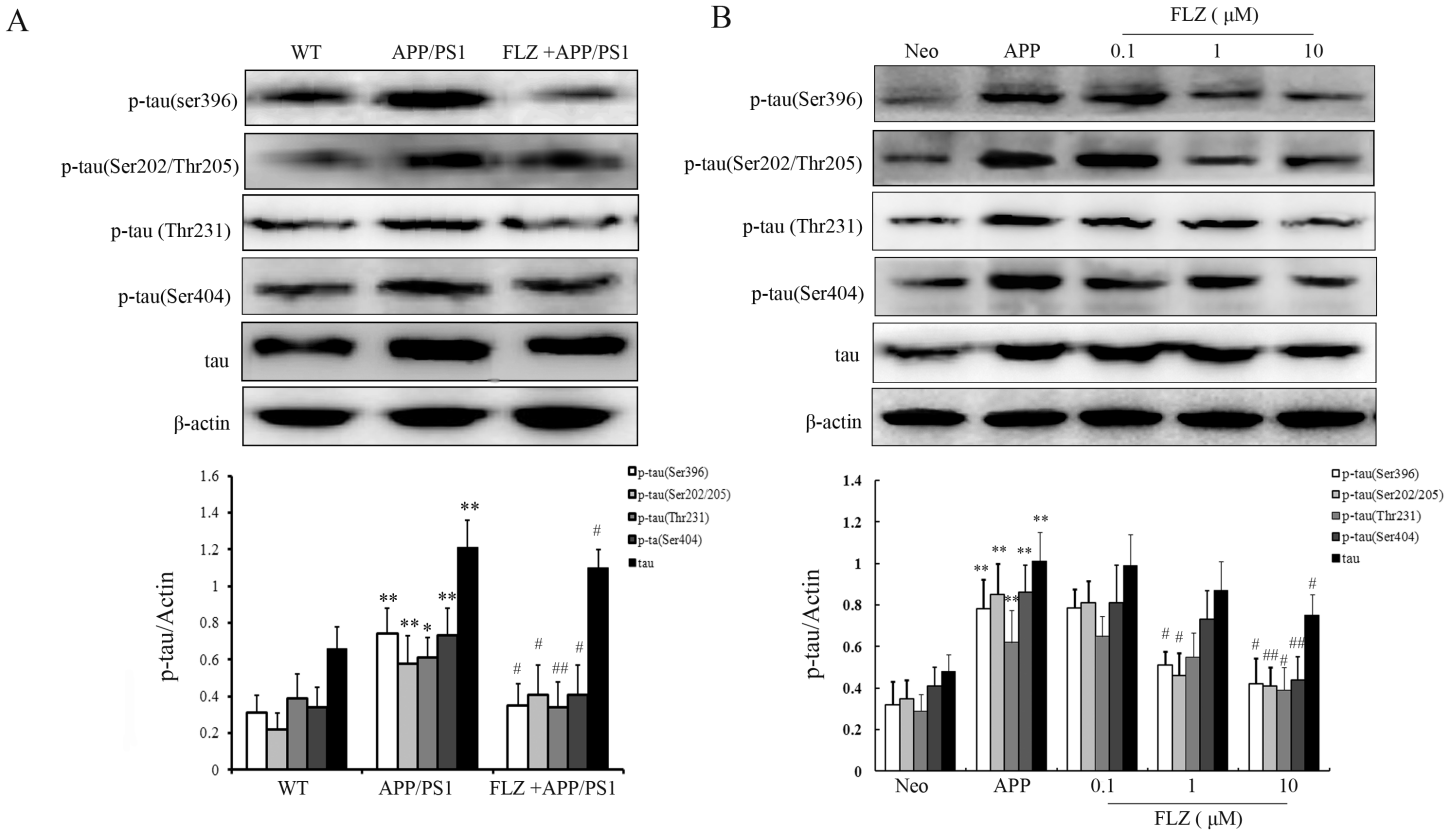
FLZ attenuated tau phosphorylation. APP/PS1 double transgenic mice were orally treated with FLZ 150 mg/kg for 20 weeks. SH-SY5Y (APPwt/swe) cells were grown in “stimulating medium” containing 50% DMEM, 50% Opti-MEM, 0.5% FBS, 200 µg/ml G418 and 10 mM butyric acid sodium salt for 12 h to induce the transgene expression. FLZ (0.1, 1 and 10 µM) were incubated with cells for 24 h. Antibodies against p-tau (Ser396), p-tau (Ser202/Thr205), p-tau (Thr231) and p-tau (Ser404) were subjected to Western blot assay. A representative immunoblot from 4 mice was shown. Results were expressed as mean±SD. (A) Western blot result in hippocampus of APP/PS1 mice. *P<0.05, **P<0.01 *vs*. WT mice; ^#^ *P*<0.05,^##^ *P*<0.01 *vs*. APP/PS1 mice. (B) Western blot result of SH-SY5Y (APPwt/swe) cells. **P<0.01 *vs*. Neo SH-SY5Y cells, ^#^ *P*<0.05, ^##^ *P*<0.01 *vs*. solvent-treated SH-SY5Y (APPwt/swe) cells.

Besides Aβ deposition, abnormally phosphorylated tau is another major neuropathological character of AD. In this study, the effects of FLZ on tau hyperphosphorylation were assessed by Western blot assay using antibodies against different phosphorylation sites of tau, including Ser396, Thr231, Ser202/Thr205 and Ser404. The results showed that FLZ significantly reduced tau phosphorylation at these phosphorylation epitopes in APP/PS1 mouse hippocampus (Ser396∶52.70% decrease, *P*<0.05;Ser202/Thr205∶60.00% decrease, *P*<0.05; Thr231∶51.43% decrease, *P*<0.0; Ser404∶43.81% decrease, *P*<0.05) and SH-SY5Y (APPwt/swe) cells (Ser396: FLZ 1 µM: 35.44% decrease; FLZ 10 µM: 46.83% decrease, *P*<0.05; Ser202/Thr205: FLZ 1 µM: 48.15% decrease, *P*<0.05; FLZ 10 µM:51.76% decrease, *P*<0.01;Thr231: FLZ 10 µM:37.10% decrease, *P*<0.05; Ser404: FLZ 10 µM: 48.84% decrease, *P*<0.01) (Fig. 7). The attenuation of tau phosphorylation induced by FLZ implies another potential protective effect of FLZ on AD pathology.

### FLZ Attenuated Tau Phosphorylation Through Akt/GSK3β Pathway

**Figure 8:**
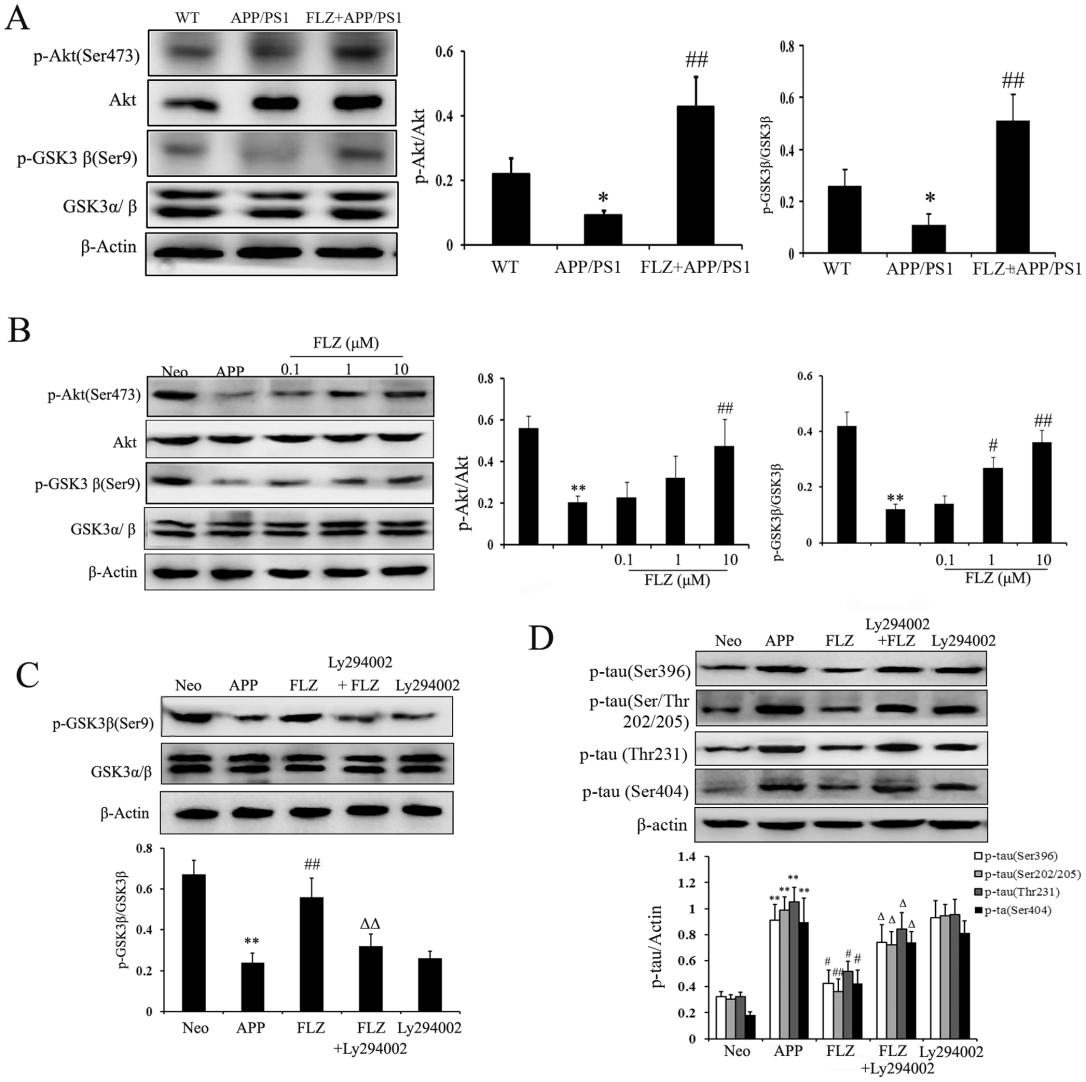
FLZ attenuated tau phosphorylation through regulating Akt/GSK3β pathway. APP/PS1 double transgenic mice were orally treated with FLZ 150 mg/kg for 20 weeks. SH-SY5Y (APPwt/swe) cells were grown in “stimulating medium” containing 50% DMEM, 50% Opti-MEM, 0.5% FBS, 200 µg/ml G418 and 10 mM butyric acid sodium salt for 12 h to induce the transgene expression. FLZ (0.1, 1 and 10 µM), Ly294002 10 µM combined with FLZ 10 µM or alone were incubated with cells for 24 h. (A) Western blot assay of p-Akt (Ser473), Akt, GSK3β and p-GSK3β (Ser9) in hippocampus of APP/PS1 mice. A representative immunoblot from 4 mice was shown. Results were expressed as mean ± SD. *P<0.05 *vs*. WT mice; ^#^ *P*<0.05, ^##^ *P*<0.01 *vs*. APP/PS1 mice. (B) Western blot assay of p-Akt (Ser473), Akt, GSK3β and p-GSK3β (Ser9) in SH-SY5Y (APPwt/swe) cells. A representative immunoblot from four independent experiments was shown. Results were expressed as mean±SD. **P<0.01 *vs*. Neo SH-SY5Y cells, ^#^ *P*<0.05, ^##^ *P*<0.01 *vs*. solvent-treated SH-SY5Y (APPwt/swe) cells. (C,D) Ly294002 was added to test the effect of Akt on GSK3β activity and tau phosphorylation in SH-SY5Y (APPwt/swe) cells treated with FLZ. A representative result of three independent experiments was shown. Results were expressed as mean±SD. **P<0.01 *vs*. Neo SH-SY5Y cells, ^#^ *P*<0.05, ^##^ *P*<0.01 *vs*. solvent-treated SH-SY5Y (APPwt/swe) cells; ^Δ^ *P*<0.05, ^ΔΔ^ *P*<0.01 *vs*. FLZ-treated SH-SY5Y (APPwt/swe) cells.

GSK3β has been reported to phosphorylate tau protein at several sites *in vitro* and *in vivo* [14]. Akt is one of the critical regulators of GSK3β, and the phosphorylation by Akt on the Ser9 residue of GSK3β leads to decreased enzymatic activity of GSK3β [15], then eventually affects tau phosphorylation. To study the mechanism underlies FLZ’s inhibitory effect on tau phosphorylation, the effect of FLZ on Akt/GSK3β signal pathway was then investigated. The results showed that FLZ treatment significantly increased the phosphorylation of Akt at Ser473, which is an indication of its activation (mouse: *P*<0.01; cell: FLZ 10 µM: *P*<0.01). The Akt activation induced by FLZ treatment was accompanied by the de-activation of GSK3β, revealed by the increased phosphorylation of GSK3β at Ser9 in APP/PS1 mice (*P*<0.01) and SH-SY5Y (APPswe/wt) cells (FLZ 1 µM: *P*<0.05; FLZ 10 µM: *P*<0.01) (Fig. 8 A, B). We also found that using Ly294002, an inhibitor of phosphoinositide 3-kinases(PI3K)/Akt pathway, the phosphorylation of GSK3β at Ser9 was markedly reduced (*P*<0.01), so was the down-regulative effect of FLZ on tau phosphorylation (Ser396: *P*<0.05; Ser/Thr202/205: *P*<0.01; Thr231: *P*<0.05, Ser404: *P*<0.05) (Fig. 8 C, D). These results suggested that FLZ might attenuate tau phosphorylation through regulating Akt/GSK3β signal pathway. Besides AKT, there are many other enzymes reported to regulate GSK3β. The study of FLZ’s effect on other enzymes involved is still undergoing.

## Discussion

Our present finding indicated that FLZ treatment significantly improved memory deficits of APP/PS1 transgenic mice and reduced apoptosis of SH-SY5Y (APPwt/swe) cells. The beneficial effects of FLZ may attribute to its inhibition of Aβ production mediated by decreasing APP processing, as well as attenuation of tau hyperphosphorylation through regulating Akt/GSK3β signal pathway. To find the time when transgenic mice start to show behavioral damage, we detected the memory deficits with Morris water maze test at the beginning (0 week), 5 weeks, 10 weeks, 15 weeks and 20 weeks during FLZ treatment. The results showed that FLZ started to show beneficial effect on memory deficits of APP/PS1 mice from 15th week (data not shown). After several times of test, we assume the mice have built memory of the water maze, and that’s the possible reason that the escape latency of WT group mice was so obviously different from that of transgenic mice at the first day of water maze training and at 20th week of FLZ treatment, which were shown in Figure 2.

The “amyloid cascade hypothesis” suggests that increased production of amyloidogenic Aβ peptide is a major cause of neuronal and synaptic loss in AD [16]. Drugs targeted at Aβ production have been proved to show beneficial effects on neural system. Although active Aβ immunotherapy was stopped because some patients developed brain inflammation, recent alternative approaches are investigated as passive immunotherapy, basing on shorter Aβ immunogens that target the N-terminus without affecting the mid-region and C-terminus of Aβ [17]. The passive immunotherapy and advanced Aβ targeted antibody showed potential therapeutic value for AD patients in clinical trials [18]. Besides the immunotherapy, some chemical synthetic compounds were also reported to decrease Aβ deposition and modify the AD progression [19]. FLZ is a new drug candidate for AD treatment, developed by our institute. Previous studies have confirmed that FLZ ameliorates learning and memory ability of AD model mice [8], [9], [20]. The application for using FLZ in clinical trials is being evaluated by China FDA right now. To better understand the mechanism of FLZ’s neuroprotective effect, we applied FLZ on APP/PS1 transgenic mice and APP transgenic cells in the present study. The administration of FLZ lasted for 20 weeks and did not cause any changes of the mouse body weight and behavior. In our pre-clinical toxicity analysis, consecutive administration of FLZ to rats for 30 days did not show any toxicity. Our data demonstrated that FLZ attenuated Aβ production both *in vitro* and *in vivo*. APP is processed by at least two distinct proteolytic pathways, the amyloidogenic and non-amyloidogenic APP processing pathway. The amyloidogenic pathway involves cleavage by BACE1, a membrane-bound aspartyl protease that generates the N terminus of the Aβ peptide. It has been shown that APP phosphorylation at Thr668 facilitates BACE1 cleavage and increases Aβ production [21]. Thus, BACE1 represents a key target protein in the development of new potential drugs for the treatment of AD. CTS-21166 is the first BACE1 inhibitor tested in clinical trials, which shows dose-related reduction in Aβ_40_ [22]. In the present study, it was showed that FLZ markedly reduced APP phosphorylation at Thr668 and BACE1 expression *in vitro* and *in vivo*, and this may be one of the mechanisms that lead to the attenuation of Aβ production by FLZ. The non-amyloidogenic pathway involves α-secretase, which cleavages APP to generate a soluble fragment of APP (sAPP) that prevents Aβ production and accumulation. Previous data from our laboratory indicated that FLZ reduced Aβ production by stimulating nonamyloidogenic sAPP production, which was mediated by increasing α-secretase ability [23]. Combining these results, we postulated that FLZ reduced Aβ production through regulating APP processing, i.e., FLZ promoted non-amyloidogenic pathway by increasing α-secretase activity and inhibited amyloidogenic pathway by decreasing BACE1 activity. Another approach to reduce Aβ deposition is to elevate its degradation. FLZ did not affect Aβ degradation, as we found FLZ has no effect on IDE, a major endopeptidase involves in Aβ enzymatic degradation. The above results indicated that regulating Aβ production might be one of the mechanisms of FLZ to treat AD.

Tau oligomer is used as a potential target for immunotherapy of AD and other tauopathies as well [24]. Compounds that inhibit tau hyperphosphorylation showed positive effects on cognitive function in clinical trials, such as AL108 [25]. Studies have shown that amyloid deposits and neurofibrillary tangles development may be related [26]. The Aβ-tau relationship is supported by the facts that there is increased tau phosphorylation in mutant APP transgenic mice, besides overexpression of Aβ peptides [27]. Furthermore, intracerebral injection of Aβ to mutant tau transgenic mice was reported to cause elevated tau phosphorylation [28]. Consistently, inhibition of Aβ accumulation markedly reduces the onset of tau pathology [29]. In the present study, we found that tau phosphorylation markedly decreased after FLZ treatment, which might at least partially be attributed to FLZ’s inhibitory effect on Aβ. Neurofibrillary tangle formation is regulated by several protein kinases, which cause phosphorylation at over 79 serine/threonine residues on tau [30]. In this study, the data demonstrated that FLZ attenuated tau hyperphosphorylation at several sites. We further investigated the enzymes that induced tau phosphorylation, and focused on GSK3β, which is an important kinase responsible for APP phosphorylation in neuronal cells [14], [31]. Many upstream kinases, such as protein kinase A (PKA), Akt, protein kinase C (PKC) and p70 ribosomal S6 kinase (p70S6K) are known to phosphorylate Ser9 of GSK3β. Among these kinases, the activity of GSK3β is negatively regulated by Akt through phosphorylation at Ser9 epitope in neurons [15]. Our present results suggested that FLZ attenuated tau hyperphosphorylation primarily through inhibition of Akt/GSK3β pathway. There are several evidences that support this hypothesis. First, FLZ treatment increased Akt activity and inhibited GSK3β activity both *in vivo* and *in vitro*. In addition, the inhibitory effect of FLZ on GSK3β was suppressed by inhibiting Akt activity. Furthermore, inhibiting the activity of Akt suppressed FLZ-induced decrease of tau phosphorylation. These data suggested that besides the indirect effect mediated by inhibiting Aβ accumulation, Akt/GSK3β pathway might be the other possible mechanism involved in the inhibitory effect of FLZ on tau hyperphosphorylation. However, how the Akt/GSK3β pathway is regulated by FLZ still needs further investigation.

The focus of the present work is the mechanism of the neuroprotective effects of FLZ on AD pathology. Our study provides the first evidence that FLZ treatment decreases APP processing by BACE1 and inhibits tau hyperphosphorylation mediated by the Akt/GSK3β pathway, highlighting the potential of FLZ as a therapy agent against AD.

## Supporting Information

File S1 **File includes Fig. S1–S3.** Figure S1. Morris water maze test of learning and memory deficits of APP/PS1 mice. (A) The latencies of mice to find the destination. (B) The number of platform crossing of mice. Results were expressed as mean ± SD.**P<0.01 vs, WT mice, n = 10 in WT group, n = 20 in APP/PS1 group. Figure S2. Body weight of APP/PS1 mice during Water maze test. Figure S3. Aβ production in cortex of APP/PS1 mice. Immunohistochemistry of Aβ deposits in cortex. Representative sections of cortex from 5 mice were shown. Results were expressed as mean ± SD. **P<0.01 vs. WT mice; ^##^P<0.01 vs. APP/PS1 mice.
(DOC)
