# Supplementary material for: FLZ Alleviates the Memory Deficits in Transgenic Mouse Model of Alzheimer’s Disease via Decreasing Beta-Amyloid Production and Tau Hyperphosphorylation
Source: PLoS One. 2013 Nov 4;8(11):e78033. doi: 10.1371/journal.pone.0078033 (PMC3817172; doi:10.1371/journal.pone.0078033)
Supplement: File S1 — File includes Fig. S1–S3. Figure S1. Morris water maze test of learning and memory deficits of APP/PS1 mice. (A) The latencies of mice to find the destination. (B) The number of platform crossing of mice. Results were expressed as mean ± SD.**P<0.01 vs, WT mice, n = 10 in WT group, n = 20 in APP/PS1 group. Figure S2. Body weight of APP/PS1 mice during Water maze test. Figure S3. Aβ production in cortex of APP/PS1 mice. Immunohistochemistry of Aβ deposits in cortex. Representative sections of cortex from 5 mice were shown. Results were expressed as mean ± SD. **P<0.01 vs. WT mice; ##P<0.01 vs. APP/PS1 mice. (DOC) [file pone.0078033.s001.doc]

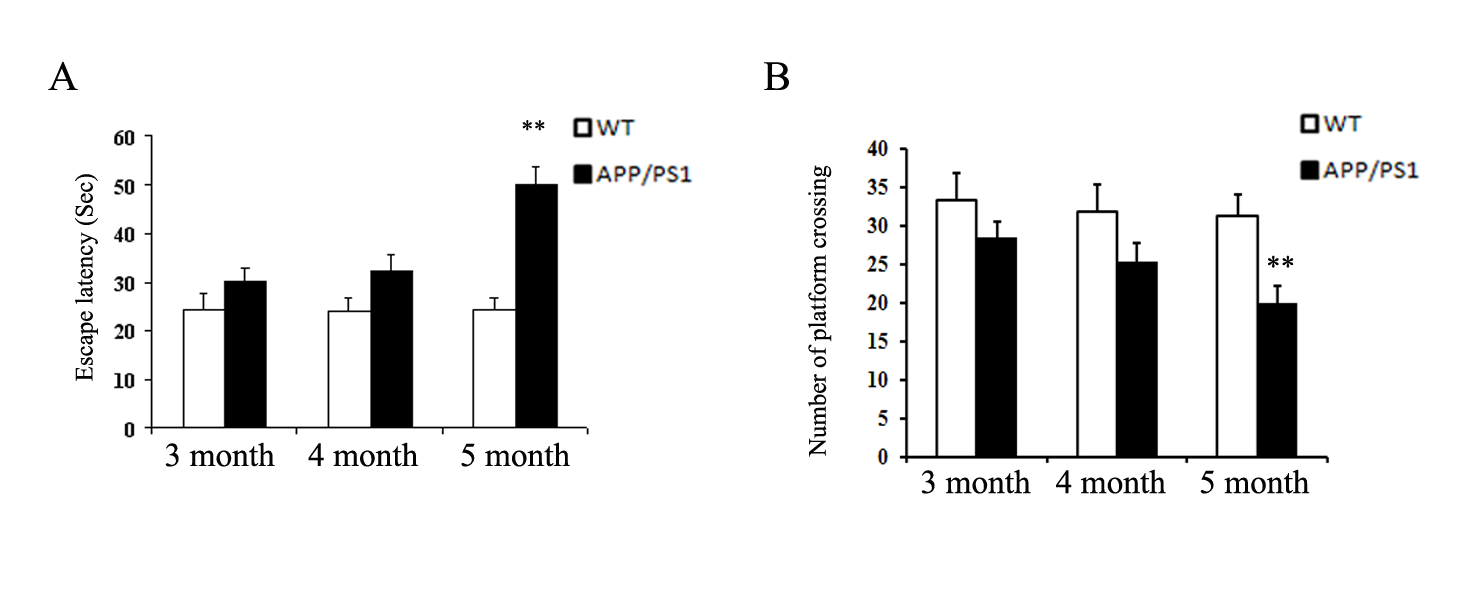


Fig. S1.Morris water maze test of learning and memory deficits of APP/PS1 mice. (A) The latencies of mice to find the destination. (B) The number of platform crossing of mice. Results were expressed as mean ± SD.^**^*P*<0.01 *vs,* WT mice, n=10 in WT group, n=20 in APP/PS1 group.


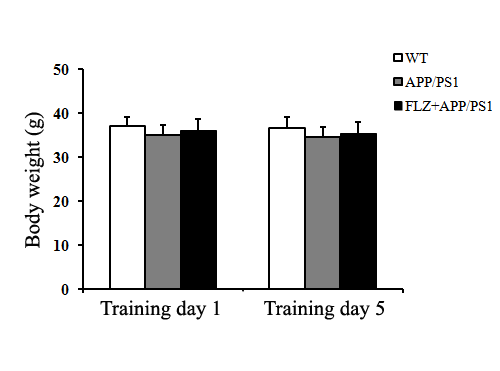


Fig.S2. Body weight of APP/PS1 mice during Water maze test.


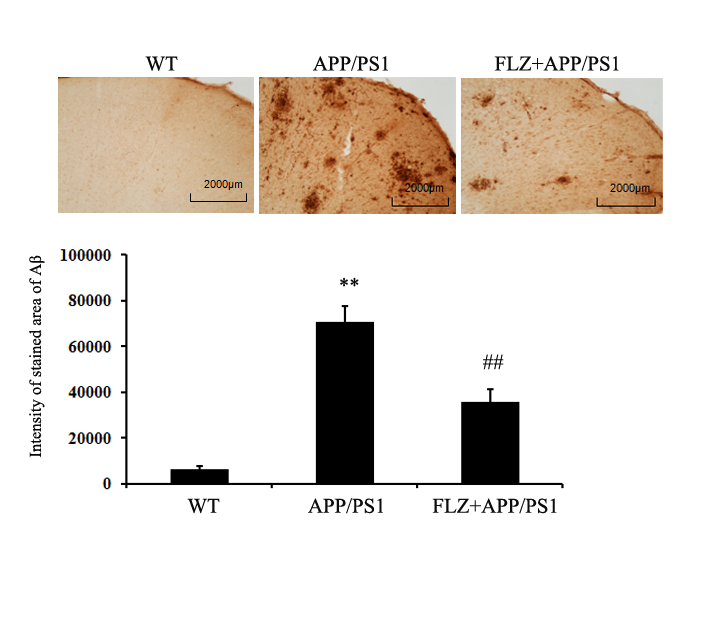


Fig. S3. Aβ production in cortex of APP/PS1 mice. Immunohistochemistry of Aβ deposits in cortex. Representative sections of cortex from 5 mice were shown. Results were expressed as mean ± SD. ^**^*P*<0.01 *vs.* WT mice; ^##^*P*<0.01 *vs.* APP/PS1 mice.
